# Callous-Unemotional Traits and Anxiety in a Community Sample of 7-Year-Olds

**DOI:** 10.1080/15374416.2013.814539

**Published:** 2013-07-23

**Authors:** Sajid Humayun, Rachel E. Kahn, Paul J. Frick, Essi Viding

**Affiliations:** Developmental Risk and Resilience Unit, University College London, Newham College University Centre, and Institute of Psychiatry, King's College London; Department of Psychology, University of New Orleans; Division of Psychology and Language Sciences, University College London

## Abstract

In forensic samples of adults and adolescents, there is evidence to suggest that there may be distinct variants of psychopathy marked by the presence/absence of significant levels of anxiety. Callous-unemotional (CU) traits can be used to characterize children who share behavioural and neurocognitive features with adult psychopaths. The aims of this paper are to (a) investigate the genetic and environmental influences on CU traits with/without anxiety and (b) explore differences in terms of concurrent and early parenting and adjustment. Discrete groups were formed on the basis of scores in the top 10% of the sample on CU and anxiety scales at age 7. Estimates of group heritability were calculated using a Defries-Fulker (DF) extremes regression model. Follow back analyses of early parenting and adjustment were conducted using multivariate analyses of covariance. There was high group heritability for CU traits with/without anxiety. Children with both high CU and anxiety showed greater levels of adjustment problems than those with CU only at age 7. The two groups did not differ in parenting characteristics. In this general population sample, evidence did not support differences in etiology for the two groups high on CU traits differing in level of anxiety.

Cleckley (1941, 1976) described psychopaths as being characterized by a superficial and manipulative interpersonal style and a profound lack of empathy/remorse. Individuals with psychopathy are typically viewed as a homogeneous grouping characterized by an absence of heightened fear and anxiety (Cleckley, 1941; Hare, 1978). However, recent research suggests that psychopathy can co-occur with elevated levels of anxiety and that the etiology of psychopathic personality may be different for those individuals with co-occuring anxiety (Karpman, 1941, 1948; Lykken, 1957, 1995; Skeem, Poythress, Edens, Lilienfeld, & Cale, 2003). Specifically, Karpman (1941, 1948) proposed a label “secondary psychopathy” to characterise individuals for whom psychopathic traits co-occur with anxiety. He suggested that “secondary psychopathy” is a product of adaptation to environmental factors such as parental rejection, abuse, or trauma. This is in contrast with “primary psychopathy,” which occurs in the absence of heightened anxiety and which Karpman proposed to be heritable in origin.

Studies of forensic adult samples suggest that individuals high on psychopathic traits without elevated anxiety show deficits in affective processing (Hiatt, Lorenz, & Newman, 2002; Newman, Schmitt, & Voss, 1997) and fear-potentiated startle response (Sutton, Vitale, & Newman, 2002) supporting the presence of a deficit in emotional responding for this group. In contrast, incarcerated adults with high levels of both psychopathy and anxiety exhibit higher rates of abuse and trauma (Blagov et al., 2011; Poythress et al., 2010), higher levels of impulsivity (Poythress et al., 2010), anger (Blagov et al., 2011), as well as reactive (Falkenbach, Poythress, & Creevy, 2008) or dating (Vidal, Skeem, & Camp, 2010) aggression than their nonanxious counterparts. These findings are consistent with the contention that this latter group shows problems in regulating emotion and behaviour related to past traumatic experiences (Skeem et al., 2003).

Recent research in adolescent forensic samples appears in agreement with the adult data. Those individuals who have high levels of both psychopathic traits and anxiety are more impulsive and show a greater history of childhood abuse and trauma than their nonanxious counterparts with high levels of psychopathy (Kimonis, Frick, Cauffman, Goldweber, & Skeem, 2012; Kimonis, Skeem, Cauffman, & Dmitrieva, 2011; Tatar, Cauffman, Kimonis, & Skeem, 2012; Vaughn, Edens, Howard, & Smith, 2009). They also show more problems with depression and anger (Kimonis et al., 2011; Kimonis et al., 2012; Lee, Salekin, & Iselin, 2010; Vaughn et al., 2009). Further, and again consistent with the research on adults, the group without high anxiety shows deficits in their processing of emotional stimuli that are not apparent in the group with both psychopathic traits and high anxiety (Kimonis et al., 2012). Thus, in forensic samples of both adults and adolescents, there appear to be two distinct groups of persons with high levels of psychopathic traits but differing on their levels of anxiety, who show a number of different characteristics, which warrant the investigation of etiologically distinct pathways to psychopathic traits.

It is important to note that CU traits are a key component of psychopathy, and they can be used to characterize children who show behavioural and neurocognitive features similar to adults high on psychopathy (Frick & Viding, 2009) and who are at an elevated risk for developing psychopathy when they reach adulthood (Lynam, Derefinko, Caspi, Loeber, & Stouthamer-Loeber, 2007). Further, prior research examining differences between primary and secondary psychopathy groups on the CU dimension of psychopathy have typically found these two groups do not differ on their level of CU traits in either adult (Blagov et al., 2011; Hicks, Markon, Patrick, Krueger, & Newman, 2004; Poythress et al., 2010; but see Vassileva, Kosson, Abramowitz, & Conrod, 2005) or adolescent (Kimonis et al., 2012) samples. Given that these two groups both show high rates of CU traits, different etiological underpinnings to primary and secondary psychopathy should also be critical for understanding children high on CU traits. However, despite the considerable body of research on CU traits in children (Frick & Viding, 2009), there is a dearth of investigations regarding potential differences between those with high levels of CU traits with and without elevated anxiety in nonforensic samples of preadolescent children. Research to date has largely focused on forensic samples that are likely overrepresented with adolescents with high levels of trauma in their histories (Ford, Chapman, Connor, & Cruise, 2012).

In a previous study of a large sample of 7-year-old twins, Viding, Blair, Moffitt, and Plomin (2005) reported that teacher-rated CU traits showed substantial heritability, consistent with findings from adolescent and adult studies (Blonigen, Carlson, Kruger, & Patrick, 2003; Kimonis et al., 2012; Poythress et al., 2010). However, these authors did not consider the possibility that heritability would be modified by the presence of high rates of anxiety, as would be suggested by theories of primary and secondary psychopathy. Thus, using the same sample reported by Viding and colleagues, our primary goal was to examine whether teacher-rated CU traits in 7-year-old twins demonstrated different genetic and environmental influences depending on the presence of elevated levels of anxiety. We also explored whether children with high levels of CU traits with/without elevated levels of anxiety differed with regard to level of harsh discipline and negative parenting at age 4 or with regard to concurrent and age 4 behavioural adjustment.

## METHOD

### Participants

Data in the study come from the 1994 and 1995 cohorts of the Twins Early Development Study (see Oliver & Plomin, 2007). Zygosity was determined using a standardised questionnaire, which has been shown to have 95% accuracy (Price et al., 2000), and 75% of the sample has subsequently been confirmed with DNA markers (Freeman et al., 2003). Twin pairs where either twin had a neurological or medical condition, including a diagnosis for Autism Spectrum Disorder, were excluded from analyses. The sample that provided data at age 7 is representative of the U.K. population in terms of ethnicity and maternal education (Harlaar, Spinath, Dale, & Plomin, 2005).

### Measures

***CU traits, anxiety, Antisocial Behaviour (AB), hyperactivity, peer problems.*** Three Antisocial Process Screening Device (APSD; Frick & Hare, 2001) and four Strengths & Difficulties Questionnaire (SDQ; Goodman, 1997) items answered by teachers were used to assess CU traits. The teacher ratings of the CU scale have acceptable internal consistency in the present sample (α = .74). Further, teacher ratings on a scale with similar items have shown to factor separately from conduct problems and to predict more severe behaviour problems in other samples of young children (Dadds, Fraser, Frost, & Hawes, 2005).^1^ Anxiety was assessed using teacher ratings on the emotional problems scale of the SDQ, which has shown sensitivity to diagnoses of anxiety disorders (Goodman, Ford, Simmons, Gatward & Meltzer, 2003) and has acceptable internal consistency in the present sample (α = .75). Teachers also provided ratings of AB, hyperactivity, and peer problems on the SDQ. Parents provided ratings of AB, hyperactivity, and peer problems when children were 4 years of age using the applicable SDQ subscales. Evidence supports the reliability and validity of these SDQ scales (Goodman, 1997; Viding et al., 2005).

***Parental negative feelings and parental harsh discipline.*** Parental feelings were assessed at age 4 using the Parent Negativity scale of the Parent Feelings Questionnaire (Deater-Deckard, 2000; Knafo & Plomin, 2006), which shows good internal consistency in the Twins' Early Development Study (TEDS) sample (age 4: α = .82). At age 7, slightly modified items were used to assess negative parental feelings toward the firstborn twin. This scale also shows good internal consistency in the TEDS sample (age 7: α = .80). Parental harsh discipline was assessed at age 4 using two questionnaire items (“give a smack or slap,” “telling off or shouting”) adapted from a semistructured interview (Deater-Deckard, Dodge, Bates, & Pettit, 1998). The Parental Harsh Discipline scale shows poor internal consistency in the TEDS sample (age 4; α = .56). At age 7, a revised version of the Parental Harsh Discipline scale that has four items was used. The Parental Harsh Discipline scale shows poor internal consistency in the present sample (age 7: α = .56).

***Socioeconomic status.*** All demographic information was obtained from the first contact booklet. An indicator of socioeconomic status (SES) was created based on a factor analysis of fathers' highest education qualification, fathers' occupational status, mothers' highest qualification, mothers' occupational status, and age of mother at birth of eldest child. A single composite was created by first standardizing these five variables and then summing them using unit weights.

### Statistical Analyses

***Formation of groups for DeFries-Fulker extremes analysis.*** For the analyses of CU traits in the CU+ group, we selected same-sex twin pairs in which at least one of the twins scored high on the CU scale (defined as 1.31 *SD* above the mean) and within a normative range on the ANX scale (defined as a score lower than 1.28 SD above the mean). Both scores correspond to the 90th percentile on each dimension. This method was used for two primary reasons. First, because the large representative sample provides a normative distribution of both CU traits and anxiety, taking elevated scores allows for age-referenced cutoffs to designate non-normative levels of these dimensions. Second, this procedure was employed in the previous Viding et al. (2005) study estimating the heritability of CU traits and the current results were designed to extend these findings. This selection criterion resulted in 627 probands (496 twin pairs: 238 monozygotic [MZ] twin pairs; 258 dizygotic [DZ] twin pairs). For the analyses of CU traits in the ANX/CU+ group, we selected same-sex twin pairs in which at least one of the twins scored high on both the CU scale (defined as 1.31 *SD* above the mean) and the ANX scale (defined 1.28 *SD* above the mean). This selection criterion resulted in 119 probands (105 twin pairs: 48 MZ twin pairs; 57 DZ twin pairs).

***The DeFries-Fulker extremes analysis.*** Estimates of group heritability and group shared environment were calculated using the DeFries-Fulker extremes analysis regression model (DeFries & Fulker, 1985), which has recently been described in detail in relation to CU (see Viding et al., 2005). Standard errors were corrected to take into account the artificial inflation of sample size (Stevenson, Pennington, Gilger, DeFries, & Gillis, 1993). All genetic analyses were based on standardized residuals that correct for gender and age.

***Formation of groups for follow-back analyses.*** The final sample used for these analyses were twins where complete teacher rated data for CU traits and ANX were available at 7 years (*N* = 3,974 twins, 48% boys). One twin of each twin pair was randomly selected in order to control for the dependent nature of the twin observations and the potentially confounding effect of selecting first or second-born twins. Only same-sexed twins were included, as the traditional DeFries-Fulker extreme analysis (DeFries & Fulker, 1985) does not incorporate opposite-sexed twins. Three discrete groups were formed on the basis of the scores on the CU scale and the ANX scale: The control group (*N* = 3,629, 92% of total sample, 46% boys) were defined as those who scored less than 1.35 *SD* above the mean on the CU scale and less than 1.49 *SD* above the mean on the ANX scale. The high-CU only group (CU+: *N* = 285, 7% of total sample, 73% boys) was defined as those who scored 1.35 *SD* above the mean on the CU scale but less than 1.49 *SD* above the mean on the ANX scale. The high-CU and high-ANX group (ANX/CU+: *N* = 49, 1% of total sample, 49% boys) was defined as those who scored 1.35 *SD* above the mean on the CU scale and 1.49 *SD* above the mean on the ANX scale. Again, these scores represent the 90th percentile for each dimension and thus designated the top 10% of scores in this large normative sample. Standardised means and SD for the CU scale and ANX scale for the three groups were as follows: controls (CU scale: *M* = −0.19, *SD* = 0.79; ANX scale: *M* = −0.24, *SD* = 0.98), CU+ (CU scale: *M* = 2.1, *SD* = 0.55; ANX scale: *M* = −0.16, *SD* = 0.64), and ANX/CU+ (CU scale: *M* = 2.08, *SD* = 0.59; ANX scale: *M* = 2.61, *SD* = 0.86).

***Follow-back analyses.*** We used multivariate analyses of covariance (MANCOVAs) in a follow-back design to examine potential differences between controls, CU+, and ANX/CU+ on assessments of early behaviour problems and early parenting characteristics. Gender and SES were included as covariates to study the potential influences on early parenting characteristics. In a separate set of analyses gender was included as a covariate to study the influences on early behaviour problems. All follow-back analyses were based on standardized residuals that correct for age differences.

***Missing data.*** Complete data on parent-rated assessments of parenting characteristics were available from 3,192 to 3,813 twins at age 4 and 7 (80% to 96% of the total sample of same-sex twins) and from 3,164 twins (79% of the sample) for parent-rated assessments of child AB, hyperactivity, and peer problems at age 4. Analyses were conducted to determine potential differences in the distribution of missing data on each of the dependent variables across the three groups. No significant differences were found. Thus observed differences between the 7-year CU+ and ANX/CU+ groups on parental characteristics and behaviour problems are unlikely explained by group differences on missing data.

## RESULTS

### Defries-Fulker Extremes Analysis

We first compared the transformed co-twin mean scores for MZ and DZ probands, which are obtained by dividing the standardized score for the MZ and DZ twin samples by the difference between the zygosity and population means. As illustrated by Table 1 the transformed mean scores in the CU+ group were .73 for MZ twins and .35 for DZ twins. In the ANX/CU+ group transformed means were .72 for MZ twins and .39 for DZ twins. Doubling the difference in the MZ and DZ co-twin means yields group heritability estimates of .76 for the CU+ group and .66 for the ANX/CU+ group. This suggests that for the CU+ group about 76% and for the ANX/CU+ group about 66% of the difference between the probands and the population can be ascribed to genetic influence.

**TABLE 1:** DeFries-Fulker Extremes Analysis of CU Traits in Children With Elevated Levels of CU Traits (CU+) and in Children With Elevated Levels and Anxiety (ANX/CU+)

	*Proband Standardized M (SD)*	*Co-Twin Standardised M (SD)*	*Transformed Co-Twin M*	*h^2^g*	*c^2^g*
CU+ (*N* = 627)					
MZ Twins	1.96 (0.57)	1.42 (.98)	.73	.75 (.58 to .92)	−0.02 (−1.22 to 2.38)
DZ Twins	2.02 (0.60)	0.71 (1.16)	.35		
ANX/CU+ (*N* = 119)					
MZ Twins	2.03 (0.63)	1.45 (0.99)	.72	.66 (.33 to .95)	.05 (−1.62 to 2.28)
DZ Twins	1.98 (0.59)	0.77 (0.84)	.39		

*Note*: CU = callous-unemotional; ANX = anxiety; MZ = monozygotic; DZ = dizygotic.

As can be seen in Table 2, the DeFries-Fulker extremes analysis confirms this high group heritability for CU traits in the CU+ group (h^2^g = .75) and in the ANX/CU+ group (h^2^g = .66). There was no significant shared environmental influence for CU traits in either group.

**TABLE 2:** Mean z Scores, *SD*, and Group Comparisons on Parent-Rated Adjustment at Age 4, Teacher-Rated Adjustment at Age 7, and Parenting Characteristics at Age 4 and 7

	*Controls (a) N = 2,899–3,625*	*CU+ (b) N = 216–284*	*ANX/CU+ (c) N = 36–48*	*Significant Effects*				
	*M (SD)*	*M (SD)*	*M (SD)*	*F*	*df*	*p*	*η*^2^_*p*_	*Bonferroni-Corrected Contrast*
Parent-Rated Adjustment, Age 4								
Multivariate				10.54	6, 6288	< .001		
AB	−0.08 (0.95)	0.46 (1.14)	0.14 (1.16)	20.38	2, 3145	< .001	.013	a < b
Hyperactivity	−0.07 (0.98)	0.37 (1.07)	0.01 (1.12)	15.72	2, 3145	< .001	.010	a < b
Peer Problems	−0.05 (0.98)	0.33 (1.07)	0.22 (1.17)	13.29	2, 3145	< .001	.008	a < b
Teacher-Rated Adjustment, Age 7								
Multivariate				166.02	6, 7900	< .001		
Conduct Problems	−0.12 (0.80)	1.19 (1.47)	2.00 (2.03)	342.06	2, 3951	< .001	.148	a < b < c
Hyperactivity	−0.10 (0.91)	1.20 (1.06)	1.28 (1.19)	228.89	2, 3951	< .001	.104	a < b, c
Peer Problems	−0.11 (0.89)	1.04 (1.23)	2.15 (1.35)	303.95	2, 3951	< .001	.133	a < b < c
Parenting Characteristics, Age 4								
Multivariate				4.55	4, 5896	< .001		
Negative Feelings	−0.01 (1.00)	0.16 (0.99)	−0.12 (1.14)	1.95	2, 2948	*ns*	.001	–
Harsh Discipline	−0.05 (0.98)	0.34 (0.97)	−0.07 (1.46)	8.89	2, 2948	< .001	.006	a < b
Parenting Characteristics, Age 7								
Multivariate				6.24	4, 7014	< .001		
Negative Feelings	−0.03 (0.99)	0.26 (0.99)	0.24 (1.23)	9.02	2, 3507	< .001	.005	a < b
Harsh Parenting	−0.04 (0.99)	0.33 (0.91)	0.28 (1.23)	10.27	2, 3507	< .001	.006	a < b

*Note*: Total sample sizes vary in each group due to missing data on some measures; differences between controls and ANX/CU+ group in parenting characteristics at age 4 and 7 were not significant. *CU*+ = CU traits without high anxiety; *ANX/CU* = CU traits with high anxiety.

### Follow-Back Analyses

First, we conducted MANCOVAs to test for group differences between controls, CU+ and ANX/CU+ on AB, hyperactivity and peer problems at 7 and 4. Because of higher levels of behaviour problems in boys we included gender as a covariate. As can be seen in Table 2 post hoc comparisons that were Bonferroni corrected for multiple comparisons showed that the CU+ and ANX/CU+ groups had higher AB, hyperactivity, and peer problems than controls at 7 (*p* < .001). At age 4 AB, hyperactivity, and peer problems were higher for the CU+ group than the controls (*p* < .001) but not for the ANX/CU+ group. CU+ and ANX/CU+ groups did not differ significantly in any indices of adjustment at age 4. However, at age 7 the ANX/CU+ had significantly higher levels of AB and peer problems than the CU+ group (*p* < .001).

Second, we conducted MANCOVAs to test for differences between groups on parenting characteristics at age 4 and 7 with gender and SES included as covariates. Bonferroni adjusted post hoc comparisons revealed more harsh parenting in the CU+ group than controls at both ages (age 4: *p* < .001; age 7: *p* < .001). Parents of the CU+ group also reported more negative parental feelings than controls (*p* < .01) at age 7. There were no differences in parenting characteristics between the CU+ and ANX/CU+ groups at either age.

## DISCUSSION

The objectives of this study were to (a) examine the relative importance of genetic and environmental influences on high levels of CU traits with/without elevated anxiety and (b) explore whether children with high levels of CU traits with/without elevated anxiety differed in the type of parenting they received or in their behavioural adjustment, either concurrently (age 7) or at age 4.

Overall, our findings were not strongly supportive of the presence of distinct etiological variants of CU traits in young children in the community. Specifically, we found that high levels of CU traits with/without high levels of anxiety were both influenced strongly by genetics with negligible effects of the shared environment. These findings are similar to a previous publication using the same sample where it was reported that CU traits were under strong genetic influence, with or without the presence of antisocial behaviour (Larsson, Viding, & Plomin, 2008). These results support a strong genetic vulnerability to the development of CU traits, whether it appears in the presence or absence of high levels of anxiety or antisocial behaviour.

Further, there were no differences in parenting characteristics between CU+ and ANX/CU+ groups both at 7 and at 4 years of age. Thus, higher levels of harsh discipline or negative parental attitudes earlier in childhood did not appear to explain why some children with high levels of CU traits also had high levels of anxiety. However, we did find significant differences between the CU+ and ANX/CU+ groups in their level of antisocial behaviour and peer problems at age 7. This pattern of findings could indicate that there may be a small group of children whose non-normative levels of CU traits and anxiety reflect two separate genetic vulnerabilities, which combine to lead to more problems in adjustment. It could also suggest that the higher levels of anxiety in some children with elevated CU traits are a consequence of the behaviour problems they experience (Frick, Lilienfeld, Ellis, Loney, & Silverthorn, 1999), rather than reflecting differences in etiology.

Our findings need to be interpreted in light of several limitations. First, our sample was a community sample that likely had very low rates of very abusive and traumatic family backgrounds, as possibly reflected by the much lower rate of children with both elevated CU traits and elevated anxiety compared to what has been found in forensic samples of adolescents (Kimonis et al., 2011; Tatar et al., 2012; Vaughn et al., 2009). Thus, a secondary variant of CU traits may not have been detected because of its very low base rate in our sample. Second, another difference with past research is that previous studies exploring trauma and abuse as potential etiological factors that may differentiate between two variants of CU traits have used scales that assess for incidents of major traumatic events, as well as records or self-reports of physical and sexual abuse (Kimonis et al., 2011; Tatar et al., 2012; Vaughn et al., 2009). The measure of harsh parental discipline used in the current study may not adequately capture the more severe forms of trauma related to a secondary variant of CU traits, and it demonstrated poor internal consistency, most likely due to its shortness. Hence these findings must be treated cautiously and as preliminary. Third, it is possible that the secondary type of CU traits may not emerge until later in development, after more extended periods of experiencing abuse or other traumatic events. However, it is important to note that measures of adjustment at age 4 were on the basis of parent, not teacher, report; this may also explain the lack of differences between groups. Fourth, although there was a different distribution of boys versus girls in the two groups (73% and 49% boys in the CU+ and ANX/CU+ groups, respectively), we did not have the power to test for potential gender differences in the findings of genetic influences given our sample size. This gender difference may be a possible avenue for future research. Fifth, our analyses did not test whether different specific genes might contribute to the etiology of CU traits in those with and without high levels of anxiety. This study was unique in testing the strength of genetic influences in the two groups of young children with high levels of CU traits, but these results need replication. In short, future research needs to study possible primary and secondary variants of CU traits using genetically informed designs and test potential differences across the two groups in a wide range of samples across different developmental stages and considering potential gender differences.

## Footnotes

1 Teacher reports were used to replicate the methodology used by Viding et al. (2005), and because there is strong evidence to support the validity of teacher's report of CU traits in young children (Dadds et al., 2005). Further, when an analogous scale was created using parent report, the internal consistency of the scale was poor (α = .45), as is often reported for CU measures. Further, when a multi-informant composite was formed taking the highest of parent or teacher ratings, the scale was still less reliable than the teacher report scale (alphas of .64 and .74, for the multi-informant and teacher ratings, respectively). Further, results using this multi-informant composite were very similar to those reported previously with the CU+ and the ANX/CU+ groups showing similar levels of heritability using the multi-informant composite (.78 vs. .71, respectively). One difference in the analyses using the multi-informant composite, however, was the finding that harsh parenting was higher in the ANX/CU group than the CU+ group.

## References

[R1] Blagov P. S., Lilienfeld S. O., Patrick C. J., Powers A. D., Phifer J. E., Venables N., Cooper G. (2011). Personality constellations in incarcerated psychopathic men. Personality Disorders: Theory, Research, and Treatment.

[R2] Blonigen D. M., Carlson S. R., Krueger R. F., Patrick C. J. (2003). A twin study of self-reported psychopathic personality traits. Personality and Individual Differences.

[R3] Cleckley H. (1941). The mask of sanity: An attempt to reinterpret the so-called psychopathic personality.

[R4] Cleckley H. (1976). The mask of sanity.

[R5] Dadds M. R., Fraser J., Frost A., Hawes D. J. (2005). Disentangling the underlying dimensions of psychopathy and conduct problems in childhood: A community study. Journal of Consulting and Clinical Psychology.

[R6] Deater-Deckard K. (2000). Parenting and child behavioral adjustment in early childhood: A quantitative genetic approach to studying family processes. Child Development.

[R7] Deater-Deckard K., Dodge K. A., Bates J. E., Pettit G. S. (1998). Multiple risk actors in the development of externalising behavior problems: Group and individual differences. Development and Psychopathology.

[R8] DeFries J. C., Fulker D. W. (1985). Multiple regression analysis of twin data. Behavior Genetics.

[R9] Falkenbach D., Poythress N. G., Creevy C. (2008). The exploration of subclinical psychopathic subtypes and the relationship with types of aggression. Personality and Individual Differences.

[R10] Ford J. D., Chapman J., Connor D. F., Cruise K. R. (2012). Complex trauma and aggression in secure juvenile justice settings. Criminal Justice and Behavior.

[R11] Freeman B., Smith N., Curtis C., Huckett L., Mill J., Craig I. (2003). DNA from buccal swabs recruited by mail: evaluation of storage effects on long-term stability and suitability for multiplex polymerase chain reaction genotyping. Behavior Genetics.

[R12] Frick P. J., Hare R. D. (2001). The antisocial process screening device.

[R13] Frick P. J., Lilienfeld S. O., Ellis M., Loney B., Silverthorn P. (1999). The association between anxiety and psychopathy dimensions in children. Journal of Abnormal Psychology.

[R14] Frick P. J., Viding E. (2009). Antisocial behavior from a developmental psychopathology perspective. Development and Psychopathology.

[R15] Goodman R. (1997). The Strengths and Difficulties Questionnaire: A research note. Journal of Child Psychology and Psychiatry.

[R16] Goodman R., Ford T., Simmons H., Gatward R., Meltzer H. (2003). Using the strengths and difficulties questionnaire (SDQ) to screen for child psychiatric disorders in a community sample. International Review of Psychiatry.

[R17] Hare R. D., Hare R. D., Schalling D. (1978). Electrodermal and cardiovascular correlates of psychopathy. Psychopathic behaviour: Approaches to research.

[R18] Harlaar N., Spinath F. M., Dale P. S., Plomin R. (2005). Genetic influences on early word recognition abilities and disabilities: A study of 7-year-old twins. Journal of Child Psychology and Psychiatry.

[R19] Hiatt K. D., Lorenz A. R., Newman J. P. (2002). Assessment of emotion and language processing in psychopathic offenders: Results from a dichotic listening task. Personality and Individual Differences.

[R20] Hicks B. M., Markon K. E., Patrick C. J., Krueger R. F., Newman J. P. (2004). Identifying psychopathy subtypes on the basis of personality structure. Psychological assessment.

[R21] Karpman B. (1941). On the need of separating psychopathy into two distinct clinical types: The symptomatic and the idiopathic. Journal of Criminal Psychopathology.

[R22] Karpman B. (1948). The myth of the psychopathic personality. American Journal of Psychiatry.

[R23] Kimonis E. R., Frick P. J., Cauffman E., Goldweber A., Skeem J. (2012). Primary and secondary variants of juvenile psychopathy differ in emotional processing. Development and Psychopathology.

[R24] Kimonis E. R., Skeem J., Cauffman E., Dmitrieva J. (2011). Are secondary variants of ‘juvenile psychopathy’ more reactively violent and less psychosocially mature than primary variants?. Law and Human Behavior.

[R25] Knafo A., Plomin R. (2006). Prosocial behavior from early to middle childhood: Genetic and environmental influence on stability and change. Journal of Personality and Social Psychology.

[R26] Larsson H., Viding E., Plomin R. (2008). Callous-unemotional traits and antisocial behavior – genetic, environmental, and early parenting characteristics. Criminal Justice and Behavior.

[R27] Lee Z., Salekin R. T., Iselin A-M. R. (2010). Psychopathic traits in youth: Is there evidence for primary and secondary subtypes?. Journal of Abnormal Child Psychology.

[R28] Lykken D. T. (1957). A study of anxiety in the sociopathic personality. Journal of Abnormal and Social Psychology.

[R29] Lykken D. (1995). The antisocial personalities.

[R30] Lynam D. R., Derefinko K. J., Caspi A., Loeber R., Stouthamer-Loeber M. (2007). The content validity of juvenile psychopathy: An empirical examination. Psychological Assessment.

[R31] Newman J. P., Schmitt W., Voss W. (1997). The impact of motivationally neutral cues on psychopathic individuals: Assessing the generality of the response modulation hypothesis. Journal of Abnormal Psychology.

[R32] Oliver B. R., Plomin R. (2007). Twins' Early Development Study (TEDS): A multivariate, longitudinal genetic investigation of language, cognition and behavior problems from childhood through adolescence. Twin Research and Human Genetics.

[R33] Poythress N. G., Edens J. F., Skeem J. L., Lilienfeld S. O., Douglas K. S., Frick P. J., Wang T. (2010). Identifying subtypes among offenders with antisocial personality disorder: A cluster-analytic study. Journal of Abnormal Psychology.

[R34] Price T. S., Freeman B., Craig I., Petrill S. A., Ebersole L., Plomin R. (2000). Infant zygosity can be assigned by parental report questionnaire data. Twin Research.

[R35] Skeem J. L., Poythress N., Edens J. F., Lilienfeld S. O., Cale E. M. (2003). Psychopathic personality or personalities? Exploring potential variants of psychopathy and their implications for risk assessment. Aggression and Violent Behavior.

[R36] Stevenson J., Pennington B. F., Gilger J. W., DeFries J. C., Gillis J. J. (1993). Hyperactivity and spelling disability: Testing for shared genetic aetiology. Journal of Child Psychology and Psychiatry and Allied Disciplines.

[R37] Sutton S. K., Vitale J. E., Newman J. P. (2002). Emotion among women with psychopathy during picture perception. Journal of Abnormal Psychology.

[R38] Tatar J. R., Cauffman E., Kimonis E. R., Skeem J. L. (2012). Victimization history and post-traumatic stress: An analysis of psychopathy variants in male juvenile offenders. Journal of Child and Adolescent Trauma.

[R39] Vassileva J., Kosson D. S., Abramowitz C., Conrod P. (2005). Psychopathy versus psychopathies in classifying criminal offenders. Legal and Criminological Psychology.

[R40] Vaughn M. G., Edens J. F., Howard M. O., Smith S. T. (2009). An investigation of primary and secondary psychopathy in a statewide sample of incarcerated youth. Youth Violence and Juvenile Justice.

[R41] Vidal S., Skeem J., Camp J. (2010). Emotional intelligence. Painting different paths for low-anxious and high-anxious psychopathic variants. Law and Human Behavior.

[R42] Viding E., Blair J. R., Moffitt T. E., Plomin R. (2005). Evidence for substantial genetic risk for psychopathy in 7-year-olds. Journal of Child Psychology and Psychiatry.

